# Exercise Capacity, Ventilatory Response, and Gas Exchange in COPD Patients With Mild to Severe Obstruction Residing at High Altitude

**DOI:** 10.3389/fphys.2021.668144

**Published:** 2021-06-18

**Authors:** Mauricio Gonzalez-Garcia, Margarita Barrero, Dario Maldonado

**Affiliations:** ^1^Pulmonary Function Testing Laboratory, Fundación Neumologica Colombiana, Bogotá, Colombia; ^2^Faculty of Medicine, Universidad de La Sabana, Bogotá, Colombia

**Keywords:** exercise, chronic obstructive pulmonary disease, altitude, cardiopulmonary exercise testing, gas exchange, hypoxia, dynamic hyperinflation, ventilatory inefficiency

## Abstract

**Background:**

Exercise intolerance, desaturation, and dyspnea are common features in patients with chronic obstructive pulmonary disease (COPD). At altitude, the barometric pressure (BP) decreases, and therefore the inspired oxygen pressure and the partial pressure of arterial oxygen (PaO_2_) also decrease in healthy subjects and even more in patients with COPD. Most of the studies evaluating ventilation and arterial blood gas (ABG) during exercise in COPD patients have been conducted at sea level and in small populations of people ascending to high altitudes. Our objective was to compare exercise capacity, gas exchange, ventilatory alterations, and symptoms in COPD patients at the altitude of Bogotá (2,640 m), of all degrees of severity.

**Methods:**

Measurement during a cardiopulmonary exercise test of oxygen consumption (VO_2_), minute ventilation (VE), tidal volume (VT), heart rate (HR), ventilatory equivalents of CO_2_ (VE/VCO_2_), inspiratory capacity (IC), end-tidal carbon dioxide tension (PETCO_2_), and ABG. For the comparison of the variables between the control subjects and the patients according to the GOLD stages, the non-parametric Kruskal–Wallis test or the one-way analysis of variance test was used.

**Results:**

Eighty-one controls and 525 patients with COPD aged 67.5 ± 9.1 years were included. Compared with controls, COPD patients had lower VO_2_ and VE (*p* < 0.001) and higher VE/VCO_2_ (*p* = 0.001), A-aPO_2_, and V_*D*_/V_*T*_ (*p* < 0.001). In COPD patients, PaO_2_ and saturation decreased, and delta IC (*p* = 0.004) and VT/IC increased (*p* = 0.002). These alterations were also seen in mild COPD and progressed with increasing severity of the obstruction.

**Conclusion:**

The main findings of this study in COPD patients residing at high altitude were a progressive decrease in exercise capacity, increased dyspnea, dynamic hyperinflation, restrictive mechanical constraints, and gas exchange abnormalities during exercise, across GOLD stages 1–4. In patients with mild COPD, there were also lower exercise capacity and gas exchange alterations, with significant differences from controls. Compared with studies at sea level, because of the lower inspired oxygen pressure and the compensatory increase in ventilation, hypoxemia at rest and during exercise was more severe; PaCO_2_ and PETCO_2_ were lower; and VE/VO_2_ was higher.

## Introduction

Chronic obstructive pulmonary disease (COPD) is the most prevalent chronic respiratory disease worldwide, even in cities located at high altitudes, and is the main cause in both men and women of the highest number of deaths and disability-adjusted life-years attributable to these chronic diseases (Caballero et al., 2008; Horner et al., 2017; Collaborators, 2020). Intolerance to exercise, desaturation, and dyspnea during exercise are common features in COPD patients that are related to quality of life and mortality (Nishimura et al., 2002; Oga et al., 2003; Casanova et al., 2008; Yoshimura et al., 2014).

In studies at sea level in patients with mild COPD, it has been shown that during exercise, compared to healthy subjects, oxygen uptake (VO_2_) and work rate (WR) are lower, and ventilatory equivalents for CO_2_ (VE/VCO_2_) and the dead space–to–tidal volume ratio (V_*D*_/V_*T*_) are higher, with similar values of partial pressure of arterial oxygen (PaO_2_) and the alveolar–arterial oxygen tension gradient (A-aPO_2_) (Elbehairy et al., 2015). In patients with more advanced COPD, alterations in VE/VCO_2_ and V_*D*_/V_*T*_ are more pronounced and are accompanied by hypoxemia, desaturation, widening of A-aPO_2_, and alveolar hypoventilation with CO_2_ retention (O’Donnell et al., 2002; Pinto-Plata et al., 2007; Neder et al., 2015).

At altitude, the barometric pressure (BP) decreases, and therefore the inspired oxygen pressure (PIO_2_) and arterial oxygen pressure (PaO_2_) also decrease. The increase in ventilation with the decrease of the arterial carbon dioxide pressure (PaCO_2_) is the main compensating mechanism that attenuates the drop in the PaO_2_ (West, 2004). In Bogotá, a city located at high altitude (2,640 m, BP 560 mm Hg), the PaCO_2_ at rest in healthy subjects decreases to approximately 33 mm Hg, and the PaO_2_ is 65 mm Hg, with values less than 60 mm Hg in the elderly (Gonzalez-Garcia et al., 2020) and even lower values in COPD patients (Gonzalez-Garcia et al., 2004).

Most of the studies evaluating ventilation and arterial blood gases (ABGs) during exercise in COPD patients have been made at sea level and in small populations of people ascending to or residents at very high altitudes. In a small sample of patients with moderate to severe COPD in Bogotá, we demonstrated lower VO_2_ at peak exercise in comparison to control subjects, alterations in ventilatory pattern, and higher hypoxemia in exercise than observed at sea level (Gonzalez-Garcia et al., 2004). The impact of Bogotá altitude on exercise capacity, dyspnea, and ABG alterations in exercise in COPD patients of all severity grades is not known. Our objective was to compare at the altitude of Bogotá, in a cardiopulmonary exercise test (CPET), exercise capacity, ABG, symptoms, and ventilatory alterations among COPD patients of all degrees of obstruction severity.

## Materials and Methods

### Subjects

This was a retrospective study in subjects referred between 2000 and 2019 to the Pulmonary Function Tests Laboratory of the Fundacion Neumologica Colombiana located in Bogotá (2,640 m) for a CPET. The institution’s research ethics committee approved the study and the use of the anonymous data sets (approval no. 202012-26004).

Chronic obstructive pulmonary disease patients had been referred to CPET for evaluation of exercise capacity, study of causes of dyspnea and exercise limitation, evaluation before pulmonary rehabilitation, or preoperative evaluation of benign extrathoracic pathologies. They were included if they had the ratio of forced expiratory volume in the first second/forced vital capacity (FEV_1_/FVC) < 0.7, clinical stability for at least 6 weeks and were residents for more than 20 years in Bogotá, to exclude acute changes due to ascent to altitude. The severity of the obstruction was classified according to the guidelines of the Global Initiative for Chronic Obstructive Pulmonary Disease (GOLD) using the post bronchodilator FEV_1_ (1: ≥80%; 2: 50–79%; 3: 30–49%, and 4: <30%) (Vogelmeier et al., 2017). Patients with permanent oxygen treatment or other respiratory diseases, chest wall disorders, and cardiovascular diseases other than cor pulmonale were excluded”. Control subjects, with normal spirometry, of the same age and sex, non-obese, non-smokers, untrained, and without a history of cardiopulmonary disease were included. The sample was taken during the same period of time in which patients with COPD were included, from subjects referred to CPET for evaluation of exercise capacity, exercise prescription, personalized medical check-ups, evaluation prior to work of high physical demand, or presurgical evaluation for benign extra thoracic diseases.

### Functional Tests at Rest

Spirometry, maximal voluntary ventilation (MVV), and inspiratory capacity (IC) at rest were performed on a V-MAX 229d (Sensormedics Inc., Yorba Linda, CA, United States). A certified 3-L syringe was used for calibration. Flows and volumes were reported according to BTPS conditions (body temperature, ambient pressure, saturated with water vapor). Spirometry was done according to the standards of the American Thoracic Society and European Respiratory Society, and Crapo reference equations were used (Crapo et al., 1981; Miller et al., 2005).

### Exercise Test

Exercise capacity was determined with a symptom-limited incremental test on a cycle ergometer. The test began with a 3-min rest period, followed by 3 min of pedaling without load, with a subsequent increase in workload every minute until the maximum tolerated level was reached (American Thoracic Society (ATS), and American College of Chest Physicians (ACCP), 2003). The increment (10–25 W) was individually selected, depending on the reported exercise tolerance and resting functional impairment. A continuous electrocardiogram record was kept. The WR, VO_2_, CO_2_ production (VCO_2_), minute ventilation (VE), VT, respiratory frequency (*f*_*R*_), heart rate (HR), end-tidal carbon dioxide tension (PETCO_2_), and VE/VCO_2_ were recorded as mean values of 30 s throughout the test. For data analysis, the average was evaluated during 3 min of rest and in the last minute of peak exercise. VO_2_ values were compared with the reference values of Hansen et al. (1984) and Wasserman et al. (2012).

Arterial blood gases were taken at rest and during peak exercise. The A-aPO_2_ was calculated using the alveolar gas equation: FIO_2_ × (BP-47) − PaCO_2_ × [FIO_2_ + (1 − FIO_2_)/RER] − PaO_2_, where FIO_2_ (inspired fraction of oxygen) = 0.2093, mean BP ∼ 560 mm Hg, and RER = measured respiratory exchange ratio. The V_*D*_/V_*T*_ was calculated with the PaCO_2_ and PETCO_2_. The anaerobic threshold (AT) was determined non-invasively using the v-slope method (American Thoracic Society (ATS), and American College of Chest Physicians (ACCP), 2003). The sensation of dyspnea and muscle fatigue during the test were assessed using the Borg scale (Borg, 1982). Because differences in exercise capacity were expected between the GOLD stages, the dyspnea score was corrected for peak VE (Neder et al., 2015). IC was measured in all COPD patients at rest and at peak exercise.

### Data Analysis

The normality of variables was tested using the Kolmogorov–Smirnov test. The mean and standard deviation or median and interquartile ranges for the quantitative variables and proportions for the qualitative variables were calculated. For the comparison of variables at rest and during exercise between control subjects and patients with COPD in all GOLD stages, the non-parametric Kruskal–Wallis test or the one-way analysis of variance (ANOVA) test was used, with the Bonferroni *post hoc* test for multiple comparisons. Two-tailed hypotheses were formulated with a significance level of less than 0.05. The statistical program SPSS version 15.0 was used.

## Results

### Subjects Characteristics

Four hundred forty-four COPD patients and 81 controls were included; 65% were men (Table 1). The mean age of the COPD patients was 67.5 ± 9.1 years and in controls 66.4 ± 4.5 years (*p* = 0.080). Body mass index (BMI) decreased and smoking increased from GOLD stage 1 to stage 4. Table 1 shows the decrease in MVV and IC, with the increase in obstruction (*p* < 0.001). Hemoglobin (Hb) values were significantly higher in the COPD patients at GOLD stages 2–4 than controls (*p* < 0.001).

**TABLE 1:** Subjects characteristics and resting functional variables in healthy controls and chronic obstructive pulmonary disease patients divided by Global Initiative for Chronic Obstructive Lung Disease (GOLD) (*n* = 525).

	Controls	Gold stage				
		1	2	3	4	*p*
Subjects	81	101	150	115	78	
Age, years	66.4 ± 4.5	69.7 ± 9.7^b^	69.3 ± 8.6^b^	66.2 ± 8.5^b^	63.5 ± 8.2^b^	<0.001
BMI, kg/m^2^	26.9 ± 2.9	27.0 ± 4.2^b^	25.8 ± 4.1^b^	24.0 ± 3.5^a,b^	22.3 ± 3.9^a,b^	<0.001
Smoking history, pack-years	–	20.0 (10.0–42.0)^b^	35.0 (22.0–50.0)^b^	40.0 (22.0–50.0)^b^	40.0 (27.0–50.0)^b^	<0.001
FVC, L	3.29 ± 0.71	3.50 ± 1.16^b^	2.99 ± 0.83^b^	2.84 ± 0.73^a,b^	2.39 ± 0.62^a,b^	<0.001
FVC, % predicted	107.3 ± 17.2	111.9 ± 15.3^b^	90.3 ± 13.2^a,b^	79.2 ± 14.0^a,b^	64.4 ± 13.6^a,b^	<0.001
FEV_1_, L	2.56 ± 0.56	2.24 ± 0.76^a,b^	1.63 ± 0.45^a,b^	1.15 ± 0.28^a,b^	0.73 ± 0.15^a,b^	<0.001
FEV_1_, % predicted	106.0 ± 17.1	92.2 ± 13.5^a,b^	63.3 ± 8.6^a,b^	40.5 ± 5.6^a,b^	24.7 ± 3.8^a,b^	<0.001
FEV_1_/FVC, %	77.9 ± 4.7	64.0 ± 5.2^a,b^	55.4 ± 8.3^a,b^	41.3 ± 8.0^a,b^	31.2 ± 5.8^a,b^	<0.001
MVV, L/min	112.5 ± 30.0	95.3 ± 35.3^a,b^	70.1 ± 22.3^a,b^	48.4 ± 13.9^a,b^	32.7 ± 9.1^a,b^	<0.001
IC, L	2.41 ± 0.63	2.29 ± 0.75^b^	2.05 ± 0.64^a,b^	1.86 ± 0.51^a,b^	1.47 ± 0.41^a,b^	<0.001
Hb, g/dL	15.4 ± 1.4	15.6 ± 1.9^b^	16.3 ± 2.0^a^	16.6 ± 1.8^a,b^	16.6 ± 2.4^a,b^	<0.001

*Values as mean ± SD, median (P_25_–P_75_) or *n* (%). *p*: one-way ANOVA or Kruskal–Wallis.*

*^*a*^*p* < 0.05 vs. controls.*

*^*b*^*p* < 0.05 vs. other GOLD stages.*

*BMI, body mass index; FVC, forced vital capacity; FEV_1_, forced expiratory volume in 1s; MVV, maximal voluntary ventilation; IC, inspiratory capacity; Hb, hemoglobin.*

### Variables in Exercise

**FIGURE 1:**
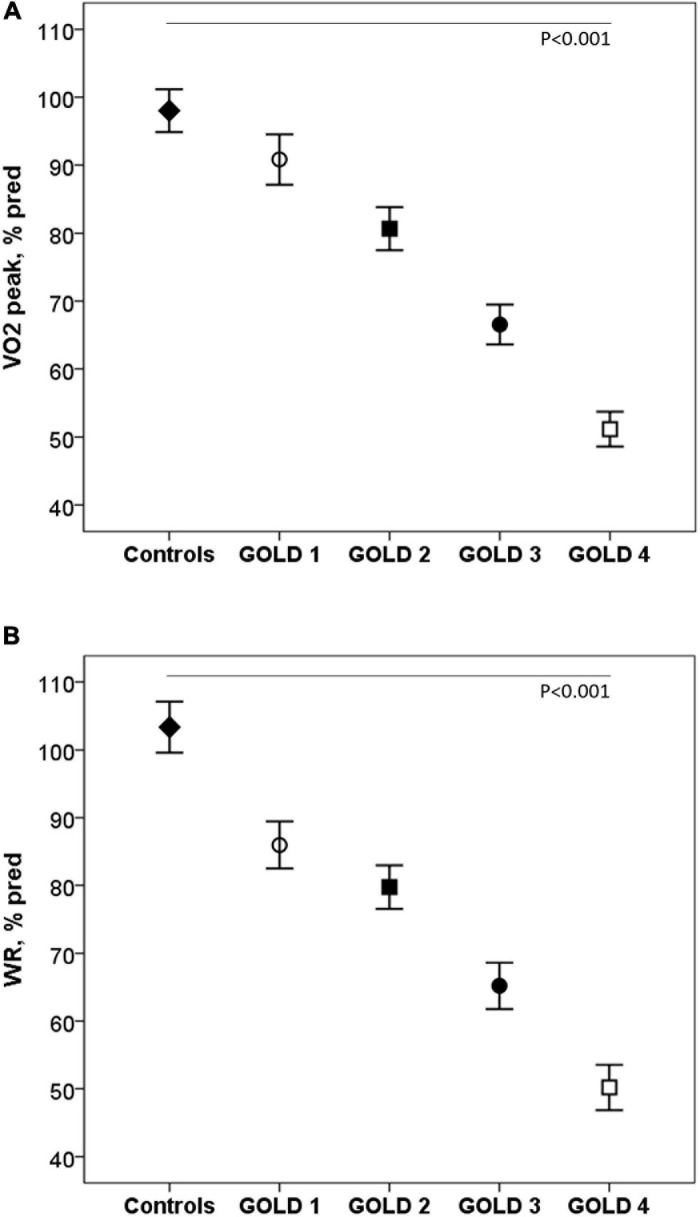
Oxygen consumption **(A)** and work rate **(B)** at peak exercise in healthy controls and COPD patients by GOLD stages. VO2: Oxygen consumption; WR: work rate; GOLD: Global initiative for Chronic Obstructive Lung Disease.◆, controls; ∘, GOLD 1; ■, GOLD 2; ∙, GOLD 3; □, GOLD 4. *P*: one-way ANOVA.

Exercise capacity decreased as the COPD severity increased. Compared with controls, COPD patients reached lower VO_2_ and workload (WR) at peak exercise, variables that progressively decreased as the COPD severity increased (*p* < 0.001) (Figure 1). The ΔVO_2_/ΔWR was lower in the patients GOLD stage 4 (*p* = 0.006). Peak HR and VO_2_/HR were also lower in COPD subjects and decreased with the severity of the obstruction (*p* < 0.001).

**FIGURE 2:**
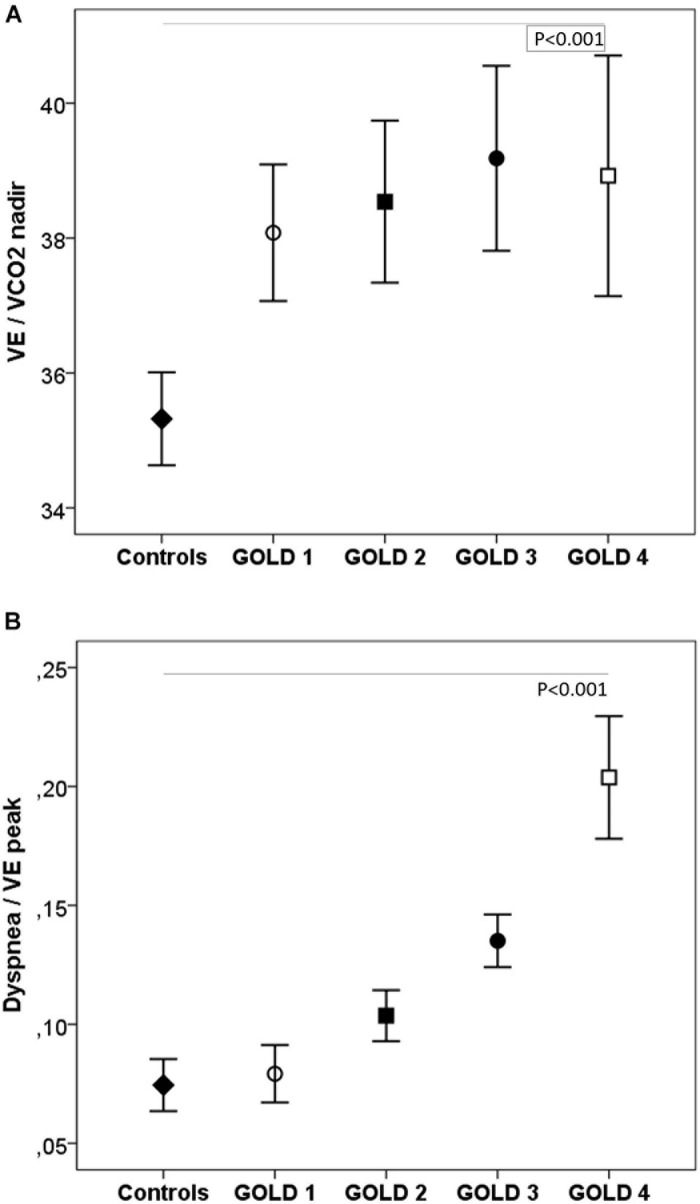
Respiratory equivalents **(A)** and dyspnea **(B)** in healthy controls and COPD patients by GOLD stage. VE, minute ventilation; VE/VCO_2_, ventilatory equivalent for carbon dioxide. ◆, controls; ∘, GOLD 1; ■, GOLD 2; ∙, GOLD 3; □, GOLD 4. *P*, one-way ANOVA.

**FIGURE 3:**
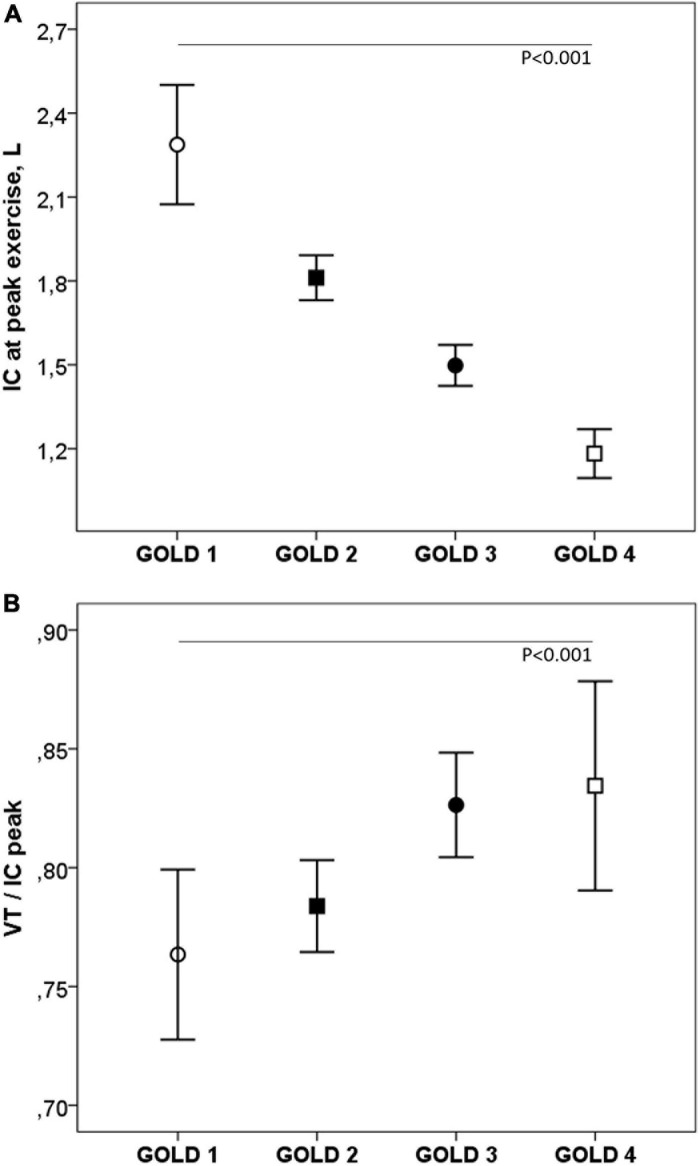
Inspiratory capacity **(A)** and VT/IC **(B)** at peak exercise in COPD patients by GOLD stage. IC, inspiratory capacity; VT, tidal volume. ∘, GOLD 1; ■, GOLD 2; ∙, GOLD 3; □, GOLD 4. *P*, one-way ANOVA.

During exercise, as the GOLD stages increased, the VE and VT were lower and there was a progressive increase in VE/MMV. VE/VCO_2_ was higher in COPD patients than in controls with no differences between the GOLD stages (Table 2 and Figure 2). At peak exercise, as the severity of the obstruction increased, the IC decreased, and the delta IC and the VT/IC increased (Figure 3).

**TABLE 2:** Peak exercise variables in healthy controls and COPD patients divided by global initiative in chronic obstructive lung disease (GOLD) (*n* = 525).

	Controls	Gold stage				
		1	2	3	4	*p*
Subjects	81	101	150	115	78	
WR, % predicted	103.4 ± 16.7	86.0 ± 16.8^a,b^	79.7 ± 17.7^a,b^	65.2 ± 15.9 ^a,b^	50.2 ± 13.5^a,b^	<0.001
VO_2_, % predicted	98.0 ± 14.2	90.8 ± 18.9^a,b^	80.7 ± 19.6^a,b^	66.6 ± 15.9^a,b^	51.2 ± 11.4^a,b^	<0.001
VO_2_ UA, % predicted	61.5 ± 14.3	60.1 ± 16.0^b^	56.8 ± 16.2^b^	50.1 ± 13.6^a^.^b^	46.4 ± 10.7^a^.^b^	<0.001
VO_2_/kg, mL/kg per min	22.1 ± 5.1	19.9 ± 5.2^a,b^	18.2 ± 5.4^a,b^	16.3 ± 3.9^a,b^	13.5 ± 3.3^a,b^	<0.001
ΔVO_2_/ΔWR, mL/min per W	11.0 ± 1.7	10.9 ± 3.3	11.8 ± 9.4^b^	10.3 ± 2.5	8.6 ± 2.5^b^	0.006
RER	1.17 ± 0.09	1.13 ± 0.12^b^	1.11 ± 0.11^a,b^	1.06 ± 0.12^a,b^	1.03 ± 0.12^a,b^	<0.001
HR, beats/min	146.2 ± 11.9	135.6 ± 17.8^b^	130.6 ± 16.8^b^	128.9 ± 17.2^b^	125.0 ± 16.6^a,b^	<0.001
HR, % predicted	87.6 ± 6.7	82.3 ± 10.3^a,b^	79.2 ± 10.1^a,b^	77.2 ± 10.0^a,b^	74.1 ± 9.6^a,b^	<0.001
O_2_pulse, mL/beat	10.5 ± 3.4	10.2 ± 3.6^b^	9.4 ± 3.1^b^	8.2 ± 2.2^a,b^	6.5 ± 1.9^a,b^	<0.001
O_2_pulse, % predicted	112.3 ± 16.7	111.7 ± 25.8^b^	103.0 ± 26.6^a,b^	86.9 ± 20.5^a,b^	69.5 ± 14.2^a,b^	<0.001
VE, L/min	65.8 ± 22.3	60.6 ± 23.0^b^	51.5 ± 16.4^a,b^	42.3 ± 11.7^a,b^	31.2 ± 8.6^a,b^	<0.001
VT, L/min	1.74 ± 0.47	1.61 ± 0.60^b^	1.39 ± 0.38^a,b^	1.22 ± 0.32^a,b^	0.96 ± 0.25^a,b^	<0.001
*f*_*R*_, rpm	37.7 ± 6.9	38.3 ± 7.2^b^	37.3 ± 7.1^b^	35.1 ± 6.5^b^	32.9 ± 6.5^a,b^	<0.001
VE/MVV	58.8 ± 11.9	65.0 ± 12.9^a,b^	74.7 ± 13.0^a,b^	88.9 ± 15.1^a,b^	97.4 ± 18.7^a,b^	<0.001
VE/VCO_2_ nadir	35.3 ± 3.1	38.1 ± 5.1	38.5 ± 7.4^a^	39.2 ± 7.4^a^	38.9 ± 7.9^a^	0.001
Delta IC, L	–	−0.41 ± 0.30^b^	−0.47 ± 0.33^b^	−0.58 ± 0.32^b^	−0.58 ± 0.35^b^	0.004
VT/IC	–	0.76 ± 0.12^b^	0.78 ± 0.12^b^	0.83 ± 0.12^b^	0.83 ± 0.15^b^	0.002
Dyspnea, Borg units	4.0 (3.0–6.0)	4.0 (3.0–5.0)^b^	5.0 (3.0–7.0)^b^	5.0 (4.0–7.0)^a,b^	5.0 (4.0–8.0)^a,b^	<0.001
Dyspnea/VE peak	0.07 ± 0.05	0.08 ± 0.06^b^	0.10 ± 0.07^a,b^	0.14 ± 0.06^a,b^	0.20 ± 0.11^a,b^	<0.001
Leg discomfort, Borg units	5.0 (3.0–7.0)	4.0 (3.0–7.0)	4.0 (3.0–5.5)	4.5 (3.0–7.0)	4.0 (2.0–5.0)^a^	<0.040
Reason for stopping exercise						<0.001
Breathing discomfort	21 (25.9)	31 (30.7)^b^	70 (46.7)^a,b^	61 (53.0)^a,b^	53 (67.9)^a,b^	
Leg discomfort	40 (49.4)	51 (50.5)^b^	49 (32.7)^b^	28 (24.3)^a,b^	12 (15.4)^a,b^	
Both	20 (24.7)	19 (18.8)	31 (20.7)	26 (22.6)	13 (16.7)	

*Values as mean ± SD, median (P_25_–P_75_) or *n* (%). *p*: one-way ANOVA or Kruskal–Wallis.*

*^*a*^*p* < 0.05 vs. controls.*

*^*b*^*p* < 0.05 vs. other GOLD stages.*

*WR, work rate; VO_2_, oxygen uptake; AT, anaerobic threshold; RER, respiratory exchange ratio; HR, heart rate; O_2_ pulse, oxygen pulse; VE, minute ventilation; VT, tidal volume; *f*_*R*_, respiratory frequency; MVV, maximal voluntary ventilation; VE/VCO_2_, ventilatory equivalent for carbon dioxide; IC, inspiratory capacity. Delta IC, exercise–rest change in IC.*

### ABGs, Dead Space, and PETCO_2_

**FIGURE 4:**
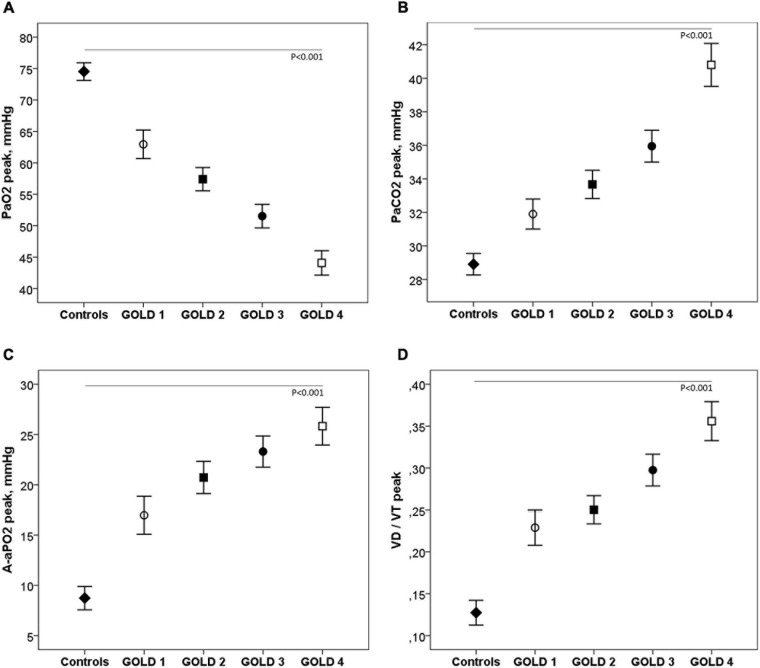
Arterial blood gases **(A–C)** and V_*D*_/V_*T*_ **(D)** at peak exercise in healthy controls and COPD patients by GOLD stage. ◆, controls; ∘, GOLD 1; ■, GOLD 2; ∙, GOLD 3; □, GOLD 4.

Table 3 shows the ABG and V_*D*_/V_*T*_ values at rest and during exercise. In patients with mild COPD during exercise, PaO_2_ and saturation were significantly lower and A-aPO_2_ and V_*D*_/V_*T*_ significantly higher than in controls. As the COPD severity increased, PaO_2_ decreased, and PaCO_2_, A-aPO_2_, and V_*D*_/V_*T*_ increased, both at rest and at peak exercise (Figure 4).

**TABLE 3:** Gas exchange parameters at rest and peak exercise in healthy controls and COPD patients divided by the global initiative in chronic obstructive lung disease (GOLD) (*n* = 525).

	Controls	Gold stage				
		1	2	3	4	*p*
**Subjects**	**81**	**101**	**150**	**115**	**78**	
**pH**						
Rest	7.44 ± 0.03	7.44 ± 0.03^b^	7.42 ± 0.03^a,b^	7.43 ± 0.04	7.42 ± 0.04^a,b^	<0.001
Peak exercise	7.35 ± 0.04	7.37 ± 0.05^b^	7.35 ± 0.05^b^	7.35 ± 0.05	7.35 ± 0.06	0.012
**PaCO_2_, mmHg**						
Rest	31.3 ± 2.4	32.3 ± 4.0^b^	33.0 ± 4.3^a,b^	33.3 ± 4.0^a,b^	36.3 ± 4.0^a,b^	<0.001
Peak exercise	28.9 ± 2.9	31.9 ± 4.5^a,b^	33.7 ± 5.2^a,b^	35.9 ± 5.1^a,b^	40.8 ± 5.6^a,b^	<0.001
**PaO_2_, mmHg**						
Rest	64.8 ± 5.2	59.1 ± 7.3^a,b^	56.0 ± 7.0^a,b^	54.7 ± 7.2^a,b^	50.1 ± 5.7^a,b^	<0.001
Peak exercise	74.5 ± 6.4	62.9 ± 11.5^a,b^	57.4 ± 11.4^a,b^	51.5 ± 10.0^a,b^	44.1 ± 8.5^a,b^	<0.001
**HCO_3_^–^, me/L**						
Rest	21.0 ± 1.6	21.7 ± 2.0^b^	21.3 ± 2.6^b^	21.9 ± 2.4^b^	23.3 ± 2.5^a,b^	<0.001
Peak exercise	16.0 ± 2.3	18.0 ± 2.8^a,b^	18.2 ± 3.0^a,b^	19.5 ± 2.6^a,b^	22.1 ± 2.9^a,b^	<0.001
**SaO_2_, %**						
Rest	92.9 ± 1.9	90.3 ± 3.6^a,b^	88.6 ± 4.3^a,b^	87.9 ± 4.6^a,b^	84.5 ± 5.1^a,b^	<0.001
Peak exercise	94.0 ± 1.7	89.0 ± 6.6^a,b^	85.7 ± 7.4^a,b^	81.6 ± 8.6^a,b^	73.6 ± 10.1^a,b^	<0.001
**A-aPO_2_, mmHg**						
Rest	7.6 ± 4.4	12.5 ± 6.1^a,b^	15.9 ± 6.4^a,b^	17.8 ± 5.8^a,b^	19.6 ± 5.6^a,b^	<0.001
Peak exercise	8.7 ± 5.3	17.0 ± 9.6^a,b^	20.7 ± 9.9^a,b^	23.3 ± 8.3^a,b^	25.8 ± 8.2^a,b^	<0.001
**V_*D*_/V_*T*_**						
Rest	0.32 ± 0.08	0.36 ± 0.11^a,b^	0.40 ± 0.08^a,b^	0.43 ± 0.08^a,b^	0.47 ± 0.07^a,b^	<0.001
Peak exercise	0.13 ± 0.07	0.23 ± 0.11^a,b^	0.25 ± 0.10^a,b^	0.30 ± 0.10^a,b^	0.36 ± 0.10^a,b^	<0.001
**Pa-ETCO_2_, mm Hg**						
Rest	1.4 ± 2.7	2.6 ± 3.2^b^	3.6 ± 3.1^a,b^	4.9 ± 3.0^a,b^	5.9 ± 3.7^a,b^	<0.001
Peak exercise	−2.2 ± 2.5	1.3 ± 4.1^a,b^	1.3 ± 3.8^a,b^	2.5 ± 4.2^a,b^	4.4 ± 5.5^a,b^	<0.001

*Values as mean ± SD. *p*: one-way ANOVA.*

*^*a*^*p* < 0.05 vs. controls.*

*^*b*^*p* < 0.05 vs. other GOLD stages.*

*PaCO_2_, partial pressure of arterial carbon dioxide; PaO_2_, partial pressure of arterial oxygen; HCO_3_^–^, bicarbonate; SaO_2_, oxygen arterial saturation; A-aPO_2_, alveolar–arterial oxygen tension gradient; V_*D*_/V_*T*_, dead space–to–tidal volume ratio; Pa-ETCO_2_, arterial end-tidal carbon dioxide pressure gradient.*

**FIGURE 5:**
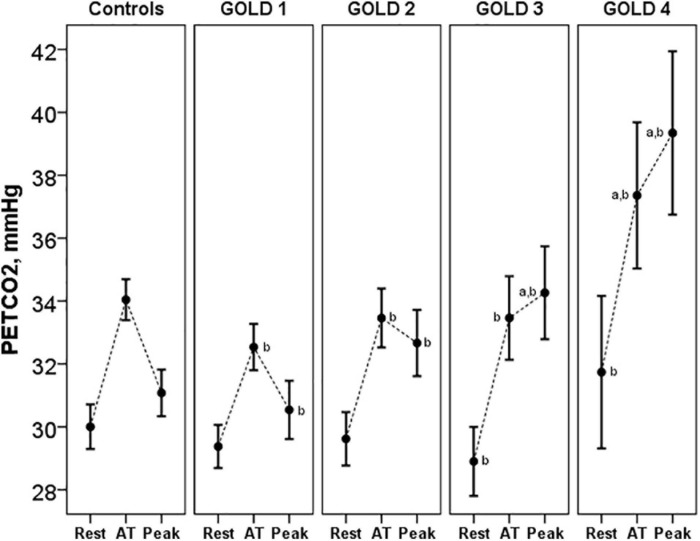
PETCO_2_ at rest, AT and peak exercise in controls and COPD patients. ^*a*^*p* < 0.05 vs. controls. ^*b*^*p* < 0.05 vs. other GOLD stages. PETCO_2_, end-tidal carbon dioxide tension; AT, anaerobic threshold.

Pa-ETCO_2_ at rest and at peak exercise was higher in COPD patients and progressively increased from GOLD stage 1 to stage 4 (Table 3). In control subjects, PETCO_2_ increased during exercise from resting values to a higher value at the AT and then decreased in peak exercise toward resting values. This PETCO_2_ trajectory was similar in COPD GOLD stage 1. In more severe patients, the PETCO_2_ at AT was higher than in controls and GOLD stage 1 patients and failed to decrease, or even raised, at peak exercise (Figure 5).

### Sensory Responses to Effort

**FIGURE 6:**
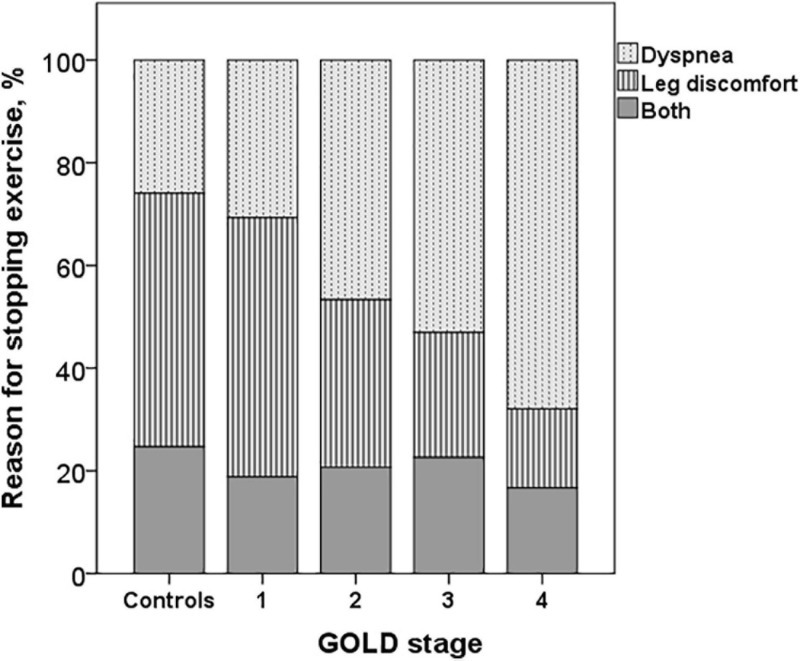
Reasons for stopping exercise in controls and COPD patients. The dyspnea increased and the leg discomfort decreased from GOLD stages 1 to 4 (*p* < 0.001).

The dyspnea at peak exercise by the Borg scale and the dyspnea adjusted to VE increased significantly from GOLD stage 1 to stage 4 (Table 2 and Figure 2). The main symptom to stop the exercise in normal subjects and in patients with mild COPD was the fatigue of the lower limbs and the dyspnea in those with more severe obstruction (Figure 6).

## Discussion

The main findings of this study, with a significant number of COPD patients residing at high altitude, were the following: (1) progressive decrease in exercise capacity, increased dyspnea, dynamic hyperinflation (DH), restrictive mechanical constraints, and gas exchange abnormalities during exercise, across GOLD stages. (2) In patients with mild COPD, there were also lower exercise capacity and gas exchange alterations, with significant differences from controls in PaO_2_, A-aPO_2_, PaCO_2_, Pa-ETCO_2_, VE/VCO_2_, and V_*D*_/V_*T*_. (3) In comparison with studies at sea level, in these patients with COPD residing at altitude, due to the lower PIO_2_, hypoxemia at rest and during exercise was more severe, and because of the compensatory increase in ventilation, PaCO_2_ and PETCO_2_ were lower, and the VE/VO_2_ ratio higher.

### Exercise Capacity

Although the progressive decrease in exercise capacity related to the severity of COPD has been previously reported, we highlight that despite hypoxemia, low saturation, and increased ventilatory requirements, VO_2_ and WR at peak exercise were similar to those described in studies at sea level, in patients of comparable age and severity of obstruction (Pinto-Plata et al., 2007; Vasilopoulou et al., 2012; Thirapatarapong et al., 2013; Neder et al., 2015), suggesting a process of adaptation to altitude in these subjects exposed to chronic hypoxia. In studies in COPD patients with acute exposure to hypoxia, ascending to an altitude similar to that of our study or using an altitude chamber, decrease in PaO_2_, SaO_2_, and PaCO_2_ at rest and in exercise has also been reported (Christensen et al., 2000; Kelly et al., 2009; Furian et al., 2018; Lichtblau et al., 2019). But contrary to our findings, in these subjects not chronically adapted to hypoxia, a significant decrease in exercise capacity has been described (Kelly et al., 2009; Furian et al., 2018).

Unlike studies that have shown a decrease in VO_2_ during exercise in healthy subjects after exposure to hypoxia for days or a few weeks (Fulco et al., 1998), relative preservation of exercise capacity has been observed in natives of the Andes and Tibet, probably related to ventilatory, circulatory, and peripheral adaptations (Favier et al., 1995; Marconi et al., 2006; Beall, 2007; Brutsaert, 2008; Calbet and Lundby, 2009). In studies in inhabitants of the Andes and Tibet, differences in adaptation to altitude have been observed, with higher levels of ventilation at rest and higher hypoxic ventilatory response (HVR) in Tibetans and higher concentrations of Hb in Andeans at the same altitude (Beall, 2007).

The ventilatory response to hypoxia varies according to the duration of the hypoxic stimulus. In healthy subjects, an acute increase in HVR has been described after ascent to altitude, and if the exposure time to hypoxia is longer (hours to months), ventilatory acclimatization to hypoxia occurs, leading to further increases in ventilation. When the exposure to hypoxia is for years or throughout life, desensitization to hypoxia arises, in which the HVR is attenuated, and both ventilation and ventilatory sensitivity to changes in PaO_2_ decrease (Chiodi, 1957; Beall et al., 1997; Pamenter and Powell, 2016). In this attenuation of HVR, genetic and physiological adaptive mechanisms are involved that determine the differences between races (Brutsaert et al., 2005; Pamenter and Powell, 2016). In Andean people, this attenuation is greater and is possibly mediated by a reduction in the chemosensitivity of peripheral receptors (Pamenter and Powell, 2016). On the other hand, studies at sea level in COPD patients have also shown increased activity and sensitivity of carotid chemoreceptors that may be related to increased cardiovascular risk (Stickland et al., 2016; Phillips et al., 2018). Although this elevated ventilatory response has not been shown to contribute to the ventilatory limitation in COPD patients at low altitude (Phillips et al., 2019), the role of HVR in high-altitude COPD patients exposed to chronic hypoxemia should be studied.

In this study, in controls and COPD patients, both at rest and during exercise, PaCO_2_ and PETCO_2_ were lower, and VE/VCO_2_ higher, compared to sea level, because of an increase in the alveolar ventilation (Chiodi, 1957; Dempsey and Forster, 1982). Also, the Hb values were higher, especially in advanced COPD stages, indicative of adaptation to altitude. In the same way, in a previous study in a large sample of healthy subjects, we also demonstrated a lower PaCO_2_ at rest and somewhat higher levels of Hb in comparison to studies at a lower altitude than Bogotá (Gassmann et al., 2019).

The administration of oxygen during exercise with correction of hypoxemia improves exercise capacity and reduces symptoms in patients with COPD at sea level (Ekstrom et al., 2016; Ward et al., 2017). In a crossover clinical trial in patients with moderate to severe COPD residing in the altitude of Bogotá, we demonstrated that the administration of oxygen during exercise significantly increased the endurance time, by reducing ventilatory demand, improving oxygen transport and cardiovascular performance (Maldonado et al., 2014). It should be noted that although with the FIO_2_ of 35% there was an increase in PaO_2_ and SaO_2_ greater than that achieved with that of 28%, this increase did not represent a significant advantage in terms of exercise duration, suggesting that the partial correction of severe hypoxemia of these patients with COPD is effective in improving the variables involved in exercise limitation.

### Cardiovascular Response

Compared to controls, HR and VO_2_/HR at peak exercise were lower in COPD subjects and decreased with the severity of the obstruction (*p* < 0.001). Although the HR at rest and during exercise has been reported to be lower at altitude, most studies have been performed after an acute exposure or a short stay at altitude (Mourot, 2018). In a study at different altitudes (sea level up to 5,100 m) in 6,289 subjects in Peru, the HR values had a minimal variation in relation to the residence altitude (Mejia et al., 2019).

Low VO_2_/HR at peak exercise suggests an abnormal hemodynamic response to exercise due to cardiovascular impairment and/or physical deconditioning (Pinto-Plata et al., 2007; Vasilopoulou et al., 2012; Thirapatarapong et al., 2013). In a study using bioimpedance, it was shown during a constant load exercise test that the greater the severity of the GOLD stage, the greater the deterioration of cardiac output (Vasilopoulou et al., 2012). Also, it has been shown that as COPD severity increases, the ventilatory mechanics is more compromised, determining alterations in the intrathoracic pressure balance, which produces a decrease in systolic volume. Other probable causes of the low oxygen pulse are the reduction of the pulmonary capillary bed and the physical deconditioning that manifests itself with physiological alterations during exercise similar to cardiovascular disease (Montes de Oca et al., 1996; O’Donnell et al., 2014).

### Ventilatory Response

As expected, the more severe the COPD, the greater the ventilatory limitation. Across GOLD stages, there was a lower increase in VE during exercise, due to a lower increase in VT. This could be explained by the progressive decrease, as the obstruction severity progressed, in baseline and peak IC, increase in delta IC due to DH, and increase in inspiratory constraints on VT expansion, shown by the progressive increase in VT/IC. Also, the VE/VVM increased with greater severity of obstruction. IC measurements in COPD patients are reproducible and useful for a more comprehensive assessment of respiratory mechanical limitations during exercise, but strategies are required to optimize results (Guenette et al., 2013; Milne et al., 2020). In these patients, IC measurements were taken at rest to familiarize them with the maneuvers, and instructions were given during exercise to achieve adequate inspiratory effort. To ensure that the respiratory pattern returned to the baseline, the graphs of the flow-volume curve were observed in real time, and the maneuvers were performed with intervals greater than 1 min.

The VE/CO_2_ nadir, a measure of ventilatory efficiency, was higher in COPD patients than in controls, but without differences between GOLD stages, similar to that described at sea level (Neder et al., 2015). The VE/VCO_2_ increase is related to different mechanisms, usually coexisting, which included mechanical ventilatory restrictions, gas exchange abnormalities, high V_*D*_/V_*T*_, enhanced chemosensitivity, and abnormal PaCO_2_ set point (Weatherald et al., 2018). In these patients with COPD residents at high altitude, we demonstrated DH, restrictive mechanical constraints, elevated V_*D*_/V_*T*_, hypercapnia, hypoxemia, and desaturation with high Pa-ETCO_2_ and A-aPO_2_. These alterations occurred even in mild COPD patients and progressively increased in more advanced stages.

The V_*D*_/V_*T*_ at rest and peak exercise was also significantly higher in COPD patients than in controls and increased across all GOLD stages. The higher physiological dead space is consistent with increased Pa-ETCO_2_, which also progressively increased from GOLD stage 1 to stage 4. The increase in Pa-ETCO_2_ was even presented in GOLD stage 1 patients, as has been shown in studies at sea level (Elbehairy et al., 2015). In relation to the PETCO_2_ trajectory, in control subjects and in patients with COPD GOLD stage 1, PETCO_2_ increased from the resting values to a higher value at the AT and then decreased at peak of exercise toward resting values, similar to previous descriptions (Hansen et al., 2007). In more severe patients, the PETCO_2_ at AT was higher than in controls and GOLD stage 1 patients and failed to decrease, or even raised, at peak exercise. These patients with more severe ventilatory impairment may not be able to increase ventilation adequately in response to acidemia and therefore have stable or increasing PETCO_2_ after AT.

PaO_2_ was significantly lower in COPD patients than in controls and progressively decreased as the severity of the obstruction increased. Although we do not have direct comparisons with data at sea level, this PaO_2_ was lower than that described in studies at sea level in COPD patients of similar age and severity of obstruction (Pinto-Plata et al., 2007). This low PaO_2_ during exercise can be explained by several mechanisms: low PIO_2_ secondary to decreased BP, low V/Q ratio, and hypercapnia (Wagner, 2015). Although in hypoxemia secondary to low PIO_2_, A-aPO_2_ should be normal and PaCO_2_ low, these two variables can be altered when there are additional mechanisms that cause hypoxemia, as is the case in patients with COPD. Low PaO_2_ during exercise was accompanied by a significant increase in A-aPO_2_ in all COPD patients, even those with mild obstruction, indicating a V/Q imbalance. PaCO_2_ during exercise was also significantly higher in all COPD patients than in controls, especially in GOLD stages 3–4, which would indicate a greater component of alveolar hypoventilation in these more severe patients. The greater PaCO_2_ seen in the more advanced stages could be explained by increased ventilation–perfusion mismatch, severe mechanical limitation with expiratory flow limitation and DH, and modifications in the respiratory controller (Montes de Oca and Celli, 1998; O’Donnell et al., 2002; O’Donnell and Laveneziana, 2006; O’Donnell et al., 2015; Poon et al., 2015). According to studies carried out at sea level with the multiple inert gas method, the diffusion limitation mechanism as a cause of hypoxemia in COPD patients is unlikely (Wagner et al., 1977; Wagner, 2015), but studies should be carried out in patients with COPD at altitude.

As significant data in this study, the A-aPO_2_ in the control subjects was similar to the values described at sea level. In studies at higher altitudes, in inhabitants of the Andes and Tibet, a low A-aPO_2_ has also been described in healthy subjects (Moore, 2017). It has been postulated that low A-aPO_2_ could preserve PaO_2_ and SaO_2_ during exercise and that these low values could be explained by a greater diffusion capacity, larger lungs and, alternatively, by a lower V/Q imbalance in these subjects (Wagner et al., 2002; Lundby et al., 2004; Calbet and Lundby, 2009).

As already mentioned, in patients with mild COPD in the altitude, there were a lower exercise capacity and gas exchange alterations, with significant differences from controls in VO_2_, WR, PaO_2_, SaO_2_, A-aPO_2_ PaCO_2_, Pa-ETCO_2_, VE/VCO_2_, and V_*D*_/V_*T*_. Studies at sea level have shown alterations in A-aPO_2_, V/Q disbalance, and high V_*D*_/V_*T*_ in patients with mild COPD at rest and during exercise (Barbera et al., 1991; Pinto-Plata et al., 2007; Rodriguez-Roisin et al., 2009; Elbehairy et al., 2015). Also, a compensatory increase in VE has been described to maintain alveolar ventilation and ABG homeostasis, leading to early mechanical limitation, exercise intolerance, and more dyspnea (Elbehairy et al., 2015; Poon et al., 2015). In contrast to this, patients with mild COPD at altitude already have a chronic compensatory increase in ventilation at rest due to the decrease in PIO_2_ and a lower PaO_2_; thus, probably the increase in ventilation during exercise is insufficient to maintain this arterial gas homeostasis.

### Sensory Responses to Effort

The main symptom for stopping exercise in normal subjects and in COPD GOLD stages 1 and 2 was fatigue of the lower limbs, and in the most severe (GOLD stages 3 and 4), it was dyspnea, as has already been described (Killian et al., 1992). In advanced stages of the disease, there is a greater decrease in IC secondary to DH, increased dead space, inefficient ventilation and mechanical constraints on VT expansion, and more severe alterations in gas exchange (O’Donnell et al., 1997, 2014, 2016; O’Donnell, 2006; Laveneziana et al., 2011; Elbehairy et al., 2015), which could explain a greater perception of dyspnea and probably an earlier interruption of exercise with less fatigue of the lower extremities. We did not find differences in dyspnea measured by the Borg scale or in the dyspnea/VE ratio at peak exercise between patients with COPD GOLD stage 1 and controls, but we did not assess dyspnea as a function of VE and WR throughout the exercise, parameters for a better evaluation of the perceptual response during exercise in these patients (O’Donnell et al., 2019; Neder et al., 2021).

This is the study conducted at altitude, with the largest number of subjects included, which assesses exercise capacity, gas exchange alterations, ventilatory limitation, and symptoms during exercise in COPD patients chronically exposed to hypoxia. Strengths of this study are the inclusion of patients of all stages of GOLD severity and a significant number of control subjects that allowed comparisons between groups. Also, the measurement of ABG and the ventilatory variables allowed us to comprehensively evaluate the limiting mechanisms of exercise in these patients. As most of the studies on the limiting factors of exercise capacity in COPD patients have been conducted at sea level and in small populations of people acutely ascending to altitude, this study in subjects chronically exposed to hypoxia increases knowledge about the pathophysiology of exercise in COPD at altitude.

Although COPD patients had to be free of exacerbations and on regular treatment to enter the study, we did not have a complete registry of medications that could modify exercise capacity in these patients. Even though we had a representative group of healthy subjects of the same age and sex, which allowed us to compare exercise capacity and ventilatory variables and ABG, we did not perform IC measurements in these subjects for comparisons with COPD patients. We also did not have carbon monoxide diffusion tests, lung volumes, and pulmonary arterial pressure data to assess the relationship of these resting functional tests with gas exchange and exercise capacity.

Taking into account that pulmonary hypertension (PH) is a common complication of COPD that affects exercise capacity (Fenster et al., 2015; Blanco et al., 2020), future studies should evaluate the impact of PH in patients with COPD at altitude. Although there are several studies in the literature that establish a relationship between mortality in COPD and some variables measured during CPET such as peak VO_2_ and respiratory equivalents (Cardoso et al., 2007; Cote et al., 2007, 2008; Oga et al., 2011; Yoshimura et al., 2014; Puente-Maestu et al., 2016; Neder et al., 2017), longitudinal studies should be carried out to evaluate the prognostic value of these variables, as well as the role of gas exchange alterations at rest and during exercise in patients with COPD living at altitude.

## Conclusion

In this study with a significant number of COPD patients and normal subjects residing at high altitude, we were able to comprehensively assess exercise capacity, symptoms, ventilatory response, and gas exchange disturbances during exercise. In patients with COPD, we observed decreased exercise capacity, increased dyspnea, DH, and gas exchange alterations, in all GOLD stages, including COPD with mild obstruction. Unlike similar studies at sea level, the degree of hypoxemia both at rest and during exercise in all degrees of severity was higher at the altitude of Bogotá.

## Data Availability Statement

The raw data supporting the conclusions of this article will be made available by the authors, without undue reservation.

## Ethics Statement

The studies involving human participants were reviewed and approved by the Comité de Ética en Investigación de la Fundación Neumológica Colombiana, Bogotá, Colombia. The ethics committee waived the requirement of written informed consent for participation.

## Author Contributions

All authors contributed to the conceptualization, design of the study, the manuscript writing, and approved the submission of the final manuscript. MG-G and MB contributed to the data abstraction and analysis. MG-G drafted the initial manuscript and the guarantor of this work.

## Conflict of Interest

The authors declare that the research was conducted in the absence of any commercial or financial relationships that could be construed as a potential conflict of interest.

## Abbreviations

COPD: chronic obstructive pulmonary disease
BP: barometric pressure
ABG: arterial blood gases
BMI: body mass index
MRCm: modified Medical Research Council scale
FVC: forced vital capacity
FEV_1_: forced expiratory volume in 1 s
MVV: maximal voluntary ventilation
PaCO_2_: partial pressure of arterial carbon dioxide
PaO_2_: partial pressure of arterial oxygen
HCO_3_^–^: bicarbonate
SaO_2_: oxygen arterial saturation
A-aPO_2_: alveolar–arterial oxygen tension gradient
V_*D*_/V_*T*_: dead space–to–tidal volume ratio
PETCO_2_: end-tidal partial pressure for carbon dioxide
Pa-ETCO_2_: arterial–end-tidal carbon dioxide pressure gradient
WR: work rate
VO_2_: oxygen uptake
VCO_2_: carbon dioxide production
RER: respiratory exchange ratio
HR: heart rate
VO_2_/HR: oxygen pulse
VE: minute ventilation
VT: tidal volume
*f*_*R*_: respiratory frequency
VE/VCO_2_: ventilatory equivalent for carbon dioxide
IC: inspiratory capacity
AT: anaerobic threshold.
